# Stretch and/or oxygen glucose deprivation (OGD) in an *in vitro* traumatic brain injury (TBI) model induces calcium alteration and inflammatory cascade

**DOI:** 10.3389/fncel.2015.00323

**Published:** 2015-08-21

**Authors:** Ellaine Salvador, Malgorzata Burek, Carola Y. Förster

**Affiliations:** Klinik und Poliklinik für Anästhesiologie, Zentrum für Operative Medizin der Universität WürzburgWürzburg, Germany

**Keywords:** blood brain barrier, cEND, astrocytes, traumatic brain injury, cell stretch, oxygen-glucose deprivation, calcium level

## Abstract

The blood-brain barrier (BBB), made up of endothelial cells of capillaries in the brain, maintains the microenvironment of the central nervous system. During ischemia and traumatic brain injury (TBI), cellular disruption leading to mechanical insult results to the BBB being compromised. Oxygen glucose deprivation (OGD) is the most commonly used *in vitro* model for ischemia. On the other hand, stretch injury is currently being used to model TBI *in vitro*. In this paper, the two methods are used alone or in combination, to assess their effects on cerebrovascular endothelial cells cEND in the presence or absence of astrocytic factors. Applying severe stretch and/or OGD to cEND cells in our experiments resulted to cell swelling and distortion. Damage to the cells induced release of lactate dehydrogenase enzyme (LDH) and nitric oxide (NO) into the cell culture medium. In addition, mRNA expression of inflammatory markers interleukin (I L)-6, IL-1α, chemokine (C-C motif) ligand 2 (CCL2) and tumor necrosis factor (TNF)-α also increased. These events could lead to the opening of calcium ion channels resulting to excitotoxicity. This could be demonstrated by increased calcium level in OGD-subjected cEND cells incubated with astrocyte-conditioned medium. Furthermore, reduction of cell membrane integrity decreased tight junction proteins claudin-5 and occludin expression. In addition, permeability of the endothelial cell monolayer increased. Also, since cell damage requires an increased uptake of glucose, expression of glucose transporter *glut1* was found to increase at the mRNA level after OGD. Overall, the effects of OGD on cEND cells appear to be more prominent than that of stretch with regards to TJ proteins, NO, *glut1* expression, and calcium level. Astrocytes potentiate these effects on calcium level in cEND cells. Combining both methods to model TBI *in vitro* shows a promising improvement to currently available models.

## Introduction

The blood-brain barrier (BBB) maintains the microenvironment of the central nervous system (CNS). Made up of endothelial cells of capillaries of the brain, it protects the brain from surging fluctuations in ion concentrations and the influx of most compounds from blood to brain. The BBB is made up of endothelial cells that make up the barrier in the apical region. On the other hand, it is ensheathed by astrocytes endfeet at the basal region. In traumatic brain injury (TBI) wherein cellular damage becomes a consequence of an extended biochemical cascade originating from an initial mechanical insult, the BBB is compromised. Breakdown of the BBB is a key feature of neuroinflammatory conditions and associated with the influx of inflammatory cells, fluids, and proteins, including complement, and cytokines.

TBI is one of the major health problems that account for high mortality worldwide. It results from an outward impact that causes immediate mechanical disruption of brain tissue and delayed pathogenic events which collectively mediate widespread neurodegeneration (Gaetz, 2004). Experimental models, both *in vivo* and *in vitro* are essential to improve outcome. Although, it is difficult to cover all the spectrum of events that might occur during TBI in one single model, an appropriate model should be able to reproduce some of these events. Several methods by which cells are injured to mimic TBI as it occurs *in vivo* exist (Thal et al., 2013). These methods may model focal or diffuse injury and include weight drop, fluid percussion, controlled cortical impact cryogenic injury, barotrauma, and acceleration/deceleration, to name a few (Morales et al., 2005; Albert-Weissenberger and Sirén, 2010). Many *in vitro* models also exist. For example, methods such as transection, compression, hydrostatic pressure, fluid shear stress, shear strain, and stretch injury are available (Kumaria and Tolias, 2008; Morrison et al., 2011). Another method recently developed in one study used a controlled scratch-induced method of injury to brain cells (mouse neuroblastoma) cultured in multi-well plates (Jowers et al., 2013). Here, we employed a technique that mimics the impact that brain cells, specifically endothelial cells of the BBB, receive during TBI. This was done by means of stretch-induced injury of our *in vitro* BBB model, the murine cerebrovascular endothelial cells cEND, a cell line which retain expression and appropriate localization of endothelial and BBB markers (Förster et al., 2005). The most currently used *in vitro* model of ischemia is oxygen glucose deprivation (OGD) of cells in culture. Ischemia generally proceeds after the occurrence of head injury.

The initial head injury results to a primary insult as an outcome of the biochemical effect of forces applied to the skull and brain manifested in milliseconds. This is usually followed by the development of secondary injury as a result of cerebral hypoxia with ischemia which leads to brain swelling and edema, activation of inflammation and blood-brain barrier leakage. Even though the period over which secondary injury occurs varies, it is generally observed that it can occur from minutes after the initial TBI to days (Gray, 2011). Any insult occurring to the brain after the initial TBI is considered a secondary injury. Cerebral ischemia or hypoxia is the ultimate cause of secondary injury after TBI (Atlee, 2007). Since this is the case *in vivo*, i.e., that secondary injury ensues after the initial TBI or head injury, an *in vitro* TBI model should be able to replicate these two succeeding events. The model presented in this paper shows the replication of these events *in vitro*.

Mechanical stress to brain microvessels as occurs in TBI quickly leads BBB disruption and dysfunction. During stretch injury, nitric oxide (NO) production and actin stress fiber formation may also be increased. This is speculated to be a mechanism modulating BBB integrity following mechanical injury. Meanwhile, tight junctions (TJs) create a paracellular barrier in endothelial cells and the proteins of the occludin and claudin family constitute the TJ strands (Förster, 2008). Expression of occludin and claudin-5 was decreased in cEND cells after administration of the inflammatory mediator tumor necrosis factor (TNF)-α (Silwedel and Förster, 2006). In addition to insult, ischemia results to a compromise in BBB integrity which leads to an increased expression of nutrient transport proteins such as those that transport glucose to the brain. GLUT-1 is a member of the facilitative glucose transporter family and is a unique transporter mediating glucose transport across the BBB (Brockmann, 2009). Metabolic demand and regional rates of cerebral glucose utilization play key roles in altering GLUT-1 expression (Xiuli et al., 2005). GLUT-1 may be upregulated at the BBB due to glutamate excitotoxicity, hypoxia, aglycemia, and mitochondrial damage (Espinoza-Rojo et al., 2010). It is also increased during hypoxia (Yu et al., 2010). In an *in vitro* experiment, OGD was shown to increase *glut1* mRNA expression (Neuhaus et al., 2012). Although information about the effects of OGD to GLUT-1 expression is available, data on the effects of stretch or a combination of stretch and OGD are scarce, if not lacking.

Mechanical injury using rapid stretch has been employed as a method to examine TBI-induced BBB disruption in the mouse brain microvessel endothelial cells bEnd3. Here, relevant injury ranges thought to occur *in vivo* during TBI stress have been used (Ellis et al., 1995; Weber et al., 1999). Meanwhile, OGD has been utilized for murine cerebral (cEND) and cerebellar (cerebEND) microvessel endothelial cells to mimic ischemia *in vitro* (Kleinschnitz et al., 2011; Neuhaus et al., 2014). Both methods have respectively been proven to be successful in replicating head injury as initial TBI event and the succeeding secondary injury such as ischemia. In this paper we evaluate the effects of stretch alone or/and in combination with OGD to the integrity of and glucose transporters expression in cEND cells as well as their effects in cEND cells co-incubated with astrocytes.

## Materials and methods

### Cell culture

Murine brain microvascular endothelial cells from the cerebral cortex (cEND) were isolated and immortalized as described previously (Förster et al., 2005; Burek et al., 2012). Briefly, cortices were isolated from 3 to 5 day-old mice, cleaned of white matter and capillary fragments, minced, and transfected with polyoma middle T oncogene. The cells were grown to confluence in tissue culture wells. These cells were then maintained in culture with mouse endothelial cell medium (Cell Biologics, Chicago, USA) supplemented with 50 U/mL penicillin/streptomycin and passaged once a week. Meanwhile, the astrocyte cell line from rat (C6 astrocytoma) was cultured using Dulbecco's Modified Eagle's Medium (DMEM) (Sigma Aldrich, USA) containing 10% fetal calf serum (FCS) and 50 U/mL penicillin/streptomycin.

### Stretch injury

Cells were grown to confluence in BioFlex 6-well culture plates with collagen-coated silastic membranes (Flexcell International Corp). Upon confluence, cells were made to differentiate in medium containing 1% fetal calf serum (FCS) for 24 h. Biaxial stretch was applied to the cells using the Cell Injury Controller II system (Virginia Commonwealth University) with 50 ms pulse duration. During cell deformation, the degree of stretch can be varied depending on the amount of pressure applied to the well. This can range from 1.8 to 4.5 psi producing 20% (5.5 mm), 35% (6.5 mm), and 55%, (7.5 mm) stretch which correspond to mild, moderate, and severe stretch, respectively (Ellis et al., 1995; Weber et al., 1999). For this study, cells were subjected only to severe stretch injury since mild and moderate stretch fail to induce LDH release in cEND cells (Salvador et al., 2013). After cell injury, cells were made to recover in the 37°C incubator for 15 and 30 min, and 1, 2, 4, 8, and 24 h. Cells that were allowed to recover for 15 min were subjected to oxygen glucose deprivation (OGD).

### Oxygen glucose deprivation (OGD)

After allowing the cells to differentiate in medium containing 1% fetal calf serum (FCS) for 24 h, the medium was replaced with DMEM lacking D-glucose. The cells were then incubated in a hypoxic incubator with the following conditions: 1% O_2_, 5% CO_2_, saturated humidity atmosphere and 37°C for 4 h as previously described (Kleinschnitz et al., 2011). After OGD, cells were either used as samples or are re-oxygenated for 20 h. Prior to re-oxygenation, cells were washed in PBS and the medium was replaced with medium containing glucose and 10% FCS.

### Cell membrane integrity

Dye exclusion is a widely used indicator of cell injury. After subjecting cells to stretch, they were stained with Image-iT® DEAD Green™ viability stain (Life Technologies), a dye that penetrates injured cells and binds to the DNA in the nuclei, producing a green fluorescence. Dye was added to the cells immediately after injury to a final concentration of 100 nm and viewed under the microscope.

### Lactate dehydrogenase (LDH) enzyme release

LDH is an enzyme released by the cell upon damage or lysis and is therefore an indicator of cell membrane integrity. The evaluation of extracellular LDH activity is a measure of cytotoxicity, since this cytosolic enzyme can be released only following the disruption of cell membranes. After cell injury, cells were made to recover in the 37°C incubator for 15 min, 30 min, 1, 2, 4, 6, 8, and 24 h and culture medium was collected at each time point. Afterwards, the cells were lysed to collect and measure total releasable LDH. The same has been done for cells subjected to OGD and a combination of stretch and OGD. Next, LDH released from the cells into the medium was measured using the LDH Cytotoxicity Detection Kit^++^ (Roche) according to manufacturer's instructions. LDH release was expressed as percentage of the total releasable LDH.

### Nitric oxide (NO) production

After subjecting the cells to stretch, 1 ml of the medium was taken from each well and ultra-filtered through a 10,000 molecular weight cut-off (MWCO) filter (Thermo Scientific). Nitric oxide (NO) production was detected by means of the Griess reaction using the Nitric Oxide (total) detection kit (Enzo Life Technologies).

### Tight junction proteins expression

Traumatic brain injury alters the expression of tight junction proteins in the cell. The mRNA expression and the protein expression levels of claudin-5 and occludin were examined.

#### Western blotting

After subjecting cells to stretch injury, they were washed twice with ice cold phosphate buffer saline (PBS) and harvested from the wells through lysis using RIPA-Buffer (50 mM Tris pH 8.0, 150 mM NaCl, 0.1% SDS, 0.5% sodium deoxycholate, 1% NP40) containing protease inhibitor cocktail (Roche) and Phenylmethylsulfonylfluoride (PMSF). After harvesting cells on ice, they were sonicated 10 × for 0.5 s on ice. Samples were then mixed with Laemmli buffer with 5% β-mercaptoethanol. They were denatured at 95°C for 5 min. Proteins were separated through 10% SDS PAGE minigels and electrophoretically wet-transferred using a Mini Trans-Blot Electrophoretic Transfer Cell (BioRad) to a PVDF membrane. Following transfer, the membrane was washed with Dulbecco's phosphate buffered saline (PBS) (Sigma Aldrich) and blocked in 5% non-fat dry milk for 1 h and incubated at 4°C overnight in 0.5% BSA-PBS containing claudin-5 (1:400), occludin (1:400) or beta actin (1:2500). Afterwards, the membrane was washed four times for 15 min each with PBS and incubated at room temperature in 1% BSA-PBS containing the secondary antibody. Next, the membrane was washed three times for 15 min each with PBS-T, incubated for 2 min in ECL solution and then imaged using Fluorchem FC2 software (Biozym) with the MultiImage II imager.

#### Quantitative real time polymerase chain reaction (qRT-PCR)

Total RNA was isolated from lysed cells and purified according to manufacturer's instructions (Nucleospin RNA II, Machery-Nagel, No. 740955). cDNA was generated using the High Capacity cDNA Reverse Transcriptase Kit (Applied Biosystems, No. 4368814). 1 μg cDNA was used for qRT-PCR with Brilliant III Ultra-Fast QPCR Master Mix (Agilent, No. 600880). The following TaqMan Gene Expression Assay primers were used: ActB (Mm01205647_g1), Cldn5 (Mm00727012_s1), Ocln (Mm00500912_m1), IL6 (Mm00446190_m1), IL1a (Mm00439620_m1), TNFa (Mm00443260_g1), CCl2 (Mm00441243_g1), glut-1 (mM01192270_m1).

### Calcium assay

The amount of calcium in the cells as affected by stretch and OGD was assessed with the Fluo-4 Direct™ Calcium Assay Kit (Invitrogen, No. F10471). Prior to stretch and OGD experiments, cEND cells were co-incubated with medium from stretched or OGD-subjected C6 cells for 24 h. Following stretch and/or OGD treatments, the cells were trypsinized, harvested, pelleted by centrifugation at 200 × g (1000 rpm) for 3 min and transferred to a 96-well plate, each sample in triplicate. 1% 5 mg/ml isradipine (Abcam Biochemicals, UK) was used as a blocker (control). The assay was conducted according to manufacturer's instructions. Fluorescence was measured 494 nm excitation and emission at 516 nm using the Tecan GENios Microplate Reader (MTX lab Systems, Inc).

### Permeability measurement

Prior to seeding the cells, the BioFlex 6-well culture plates (Flexcell International Corp) were coated with 3 ml of 0.25 mg/ml biotin conjugated-gelatin overnight then washed twice with PBS (Dubrovskyi et al., 2013). After stretch and OGD experiments, permeability was measured through fluorescein isothiocyanate (FITC)-avidin assay. Briefly, 25 μg/ml FITC-avidin was added to cells for 3 min. Unbound FITC-avidin was removed by washing twice with PBS. Next, the elastic bottoms of the wells were excised with a scalpel and mounted onto polystyrene 6-well plates. The membranes were then covered with 1 ml of PBS. Finally, the fluorescence was measured using a multilabel plate reader.

### Statistical analysis

Data were analyzed through Sigma Plot (Systat Software Inc, California) using ANOVA with pairwise comparison, Holm-Sidak method.

## Results

### Cell stretch destroys cell membrane integrity

To assess the effects of stretch in brain endothelial cell, murine cerebrovascular endothelial (cEND) cells were grown on collagen pre-coated 6-well flexible bottomed culture plates (57.75 cm^2^) and subjected to stretch injury using the cell stretcher device. Afterwards, they were examined under the microscope for morphological effects of stretch-induced injury.

**Figure 1:**
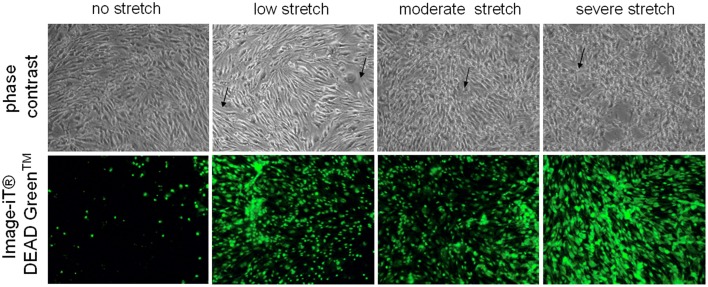
**Microscopic examination of unstretched and stretched cEND cells**. Under the light microscope, unstretched cEND cells are compact and spindle-shaped while severely stretched cells appear swollen, deformed, and retracted (indicated by arrows). Meanwhile, fluorescence microscopy shows that more cells take up the viability stain with increased stretch. Magnification 100X.

Control cells which were not subjected to injury appear as regularly shaped cEND cells without any indication of cell swelling or distortion (Figure 1). On the other hand, deformation could be observed for cells subjected to low, moderate, and severe stretch (Figure 1). After the cells were stretched with a peak pressure between 3.5 and 4.5 psi corresponding to severe stretch, they appeared markedly retracted, swollen, and deformed with notable intercellular spaces (Figure 1). Moreover, when viability stain (100 nm final concentration) was applied to the cells, the dye was excluded from most of the control cells, thus, only a few of the cells were stained (Figure 1). However, most of the cells that were subjected to stretch took up the dye (Figure 1). The viability stain used is a dye impermeant to healthy cells that becomes permeant when the plasma membrane integrity of the cells is compromised.

### Cell stretch leads to increased LDH secretion

**Figure 2:**
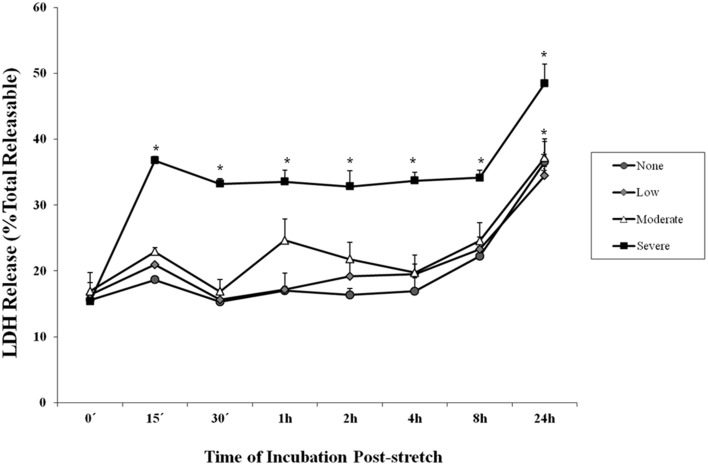
**Lactate dehydrogenase (LDH) enzyme release into the cell culture medium after stretch injury**. LDH released into the culture medium was measured at various time points after stretch induced injury. LDH was expressed as a percent of the total releasable LDH (LDH in media plus cells). Data are mean values ± SEM. The *n* for every time point is 5. Severely stretched cEND cells released a significantly greater amount of LDH as compared to unstretched cells at all time points (^*^p < 0.05).

Lactate dehydrogenase is an enzyme used to indicate the occurrence of cytotoxicity in cells. The release of LDH in the supernatant of the cell culture medium indicates cell damage. It was observed among cells subjected to severe stretch that the amount of LDH released by the cells into the cell culture medium significantly increased immediately (0 min), 15, and 30 min, 1, 2, 4, 8, and 24 h after stretch injury (Figure 2) as compared to cells that were not subjected to stretch or were subjected to low and moderate stretch (*p* < 0.05). Moreover, cells that were stretched (low, moderate, and severe) and allowed to recover for 24 h post-injury released significantly higher amount of LDH compared to other time points.

**Figure 3:**
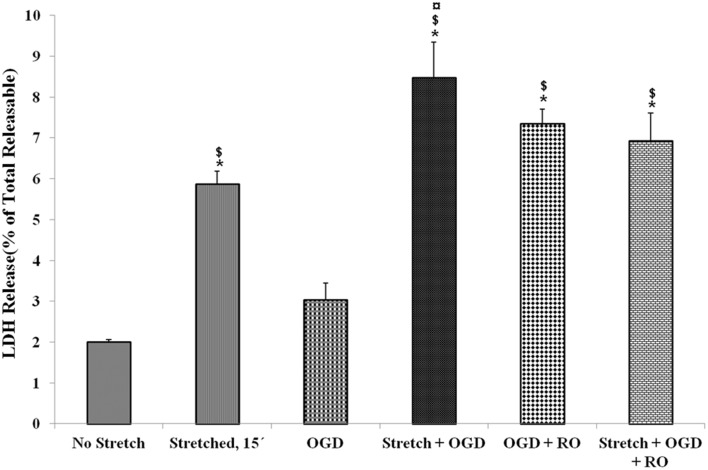
**Lactate dehydrogenase (LDH) enzyme release into the cell culture medium in the various treatment conditions**. LDH generated after stretch, OGD, a combination of both and successive reoxygenation was measured. LDH was expressed as a percent of the total releasable LDH (LDH in media plus cells). Data are mean values ± SEM, *n* = 3. *, ¤, $ *p* < 0.05 compared to no stretch, stretched, and OGD, respectively. OGD, oxygen glucose deprivation; RO, re-oxygenation.

LDH release of cells that were subjected to severe stretch and made to recover for 15 min was compared to cells subjected to OGD and cells that underwent both conditions. It was observed that stretch released significantly higher LDH compared to no stretch control as well as OGD. Meanwhile, the combination of stretch and OGD generated a significantly higher amount of LDH compared to no stretch control, stretched cells, and cells subjected to OGD (Figure 3).

### Stretch and OGD in cEND cells induce inflammatory cascade and alter NO secretion

**Figure 4:**
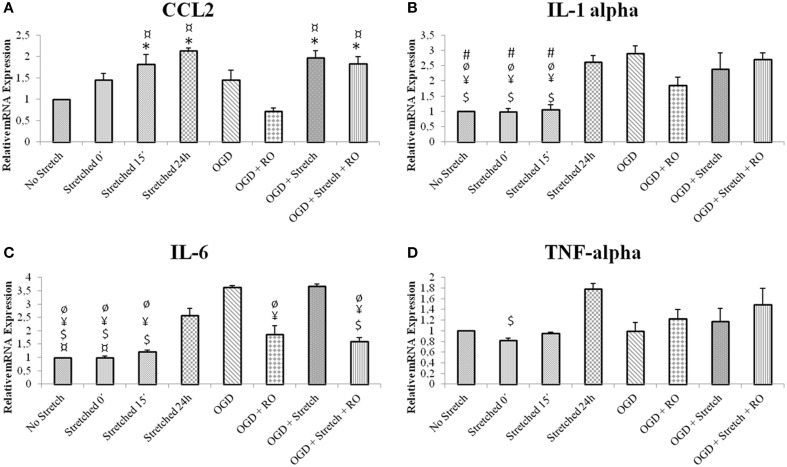
**mRNA expression of inflammatory markers in stretched cEND cells**. Values are the means (± SEM) of 3 independent experiments. Statistical significance was evaluated using One-Way ANOVA (Holm-Sidak method) *, $, ¥, ¤, ø, # *p* < 0.005 compared to no stretch, stretched 24 h, OGD, OGD + Stretch, OGD + RO, OGD + Stretch + RO, respectively. OGD, oxygen glucose deprivation; RO, re-oxygenation. **(A–D)** Selected inflammatory markers involved in TBI and ischemia.

In order to assess the characteristics of inflammatory response in our *in vitro* model, inflammatory cytokine production in cEND cells was assessed at the mRNA level. cEND cells have been previously shown to secrete various chemokines into the cell culture medium (Burek et al., 2014). IL6 is believed to be among the best markers of disease severity in patients with systemic inflammation from whatever cause (Reinhart et al., 2002). Results show that greatest mRNA expression of inflammatory markers interleukin (IL)-6, IL-1α, chemokine (C-C motif) ligand 2 (CCL2) and tumor necrosis factor (TNF)-α in cells subjected to stretch was after 24 h (*p* < 0.005). Stretched cells induced CCL2 and TNF-α expression greater than those subjected to OGD (*p* < 0.005); whereas those subjected to OGD induced greater IL1-α and IL-6 expression than the stretched cells (*p* < 0.005) (Figure 4).

**Figure 5:**
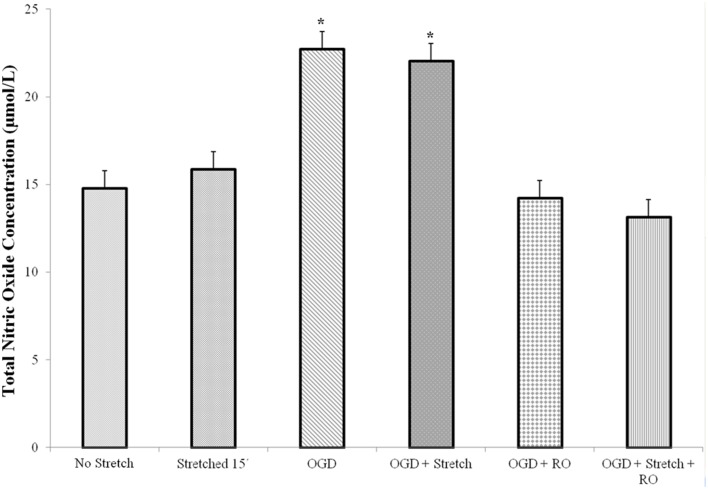
**Total nitric oxide (NO) production in cEND cells**. Values are the means (± SEM) of 3 independent experiments. Statistical significance was evaluated using One-Way ANOVA (Holm-Sidak method), ^*^p < 0.005 compared to all the rest of the treatments. OGD, oxygen glucose deprivation; RO, re-oxygenation.

Nitric oxide (NO) is a major mammalian secretory product that initiates host defense and accumulates in the brain after injury. We detected total NO production in the cells. Results of the assay showed that a significantly higher amount of NO is produced by cEND cells subjected to OGD and OGD combined to stretch compared to all other treatments (*p* < 0.005) (Figure 5).

### Stretch and OGD induce decrease of tight junction proteins expression and increase in glut-1 level

**Figure 6:**
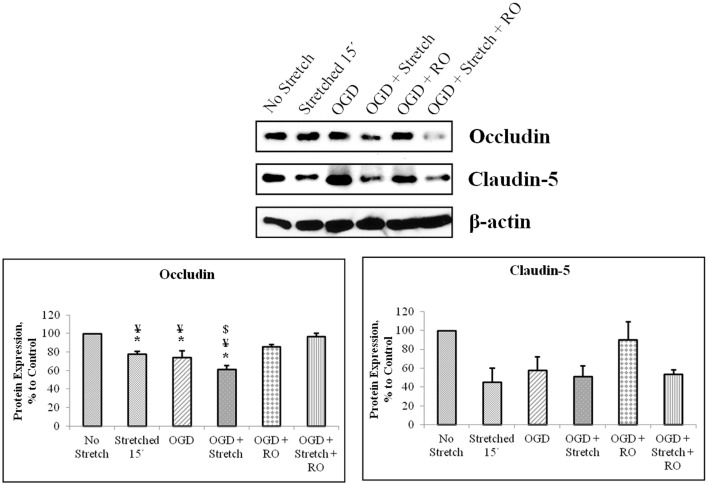
**Western blot and densitometric analyses of tight junction proteins in cEND cells**. Values are the means (± SEM) of 4–5 independent experiments normalized to β-actin. Statistical significance was evaluated using One-Way ANOVA (Holm-Sidak method), *, ¥, $ *p* < 0.005 compared to no stretch control, OGD + Stretch + RO, and OGD + RO, respectively. OGD, oxygen glucose deprivation; RO, re-oxygenation.

**Figure 7:**
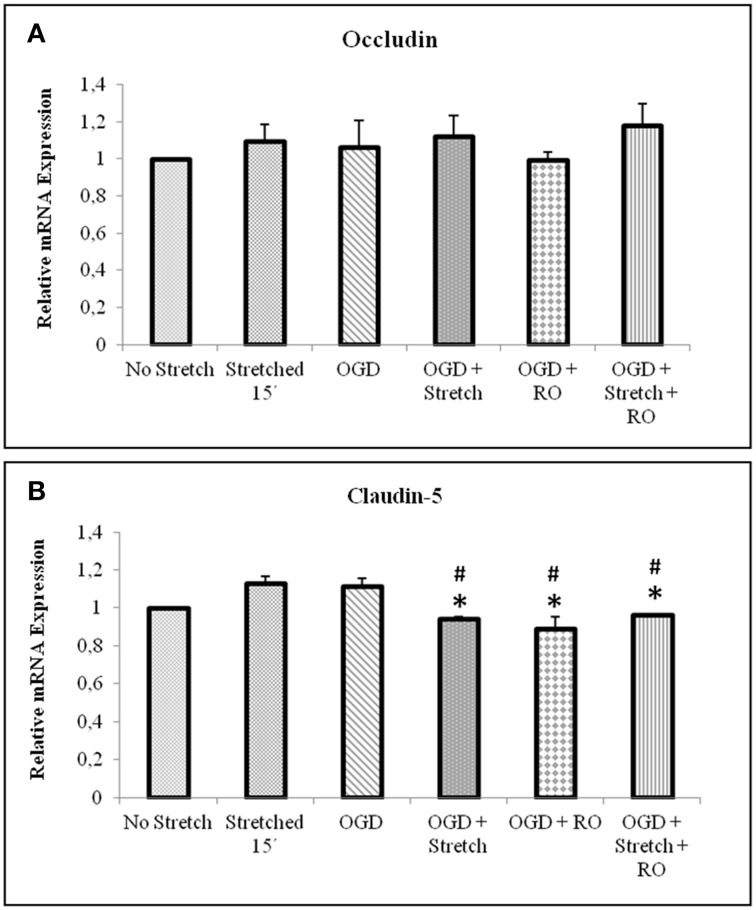
**qPCR analysis of tight junctions mRNA expression in cEND cells**. Values are the means (± SEM) of 6 independent experiments normalized to β-actin. Statistical significance was evaluated using One-Way ANOVA (Holm-Sidak method), *, # *p* < 0.005 compared to stretched 15′ and OGD, respectively. OGD, oxygen glucose deprivation; RO, re-oxygenation.

Tight junction proteins are important in the maintenance of blood-brain barrier (BBB) permeability. Once the BBB is compromised or damaged, the expression of tight junction proteins changes. Therefore, we analyzed the expression of occludin and claudin-5 both at the protein and mRNA levels. Western blot and subsequent densitometric analyses show that occludin protein expression significantly decreased (*p* < 0.005) after subjecting cells to stretch as well as to OGD and a combination of both in comparison to no stretch control. On the other hand, claudin-5 protein expression decreased after subjecting cells to stretch as well as OGD, a combination of both, and with subsequent re-oxygenation (RO) compared to control but not significantly (Figure 6). Meanwhile, qPCR results show no change in the mRNA expression of occludin among the various treatments. In contrast, mRNA expression of claudin-5 in cells subjected to a combination of OGD and stretch, OGD with subsequent RO, and OGD combined with stretch and subsequent RO decreased significantly (*p* < 0.005) compared to cells subjected to stretch and made to recover for 15 min and OGD (Figure 7).

**Figure 8:**
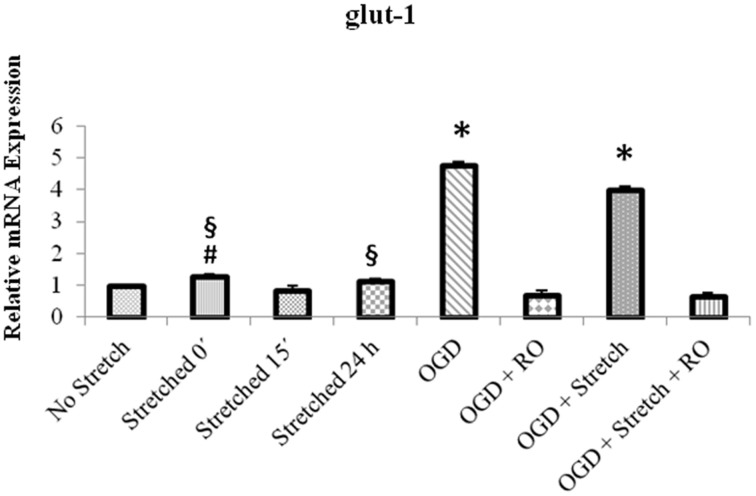
**qPCR analysis of ***glut1*** mRNA expression in cEND cells**. Values are the means (± SEM) of 4 independent experiments normalized to β-actin. Statistical significance was evaluated using One-Way ANOVA (Holm-Sidak method), *, #, § *p* < 0.005 compared to all, OGD + RO, and OGD + Stretch + RO, respectively. OGD, oxygen glucose deprivation; RO, re-oxygenation.

Quantitative real time polymerase reaction (qPCR) results show that the mRNA expression of *glut1* significantly increased in cEND cells during OGD as well as a combination of OGD and stretch (*p* < 0.005) compared to all other treatments (Figure 8).

**Figure 9:**
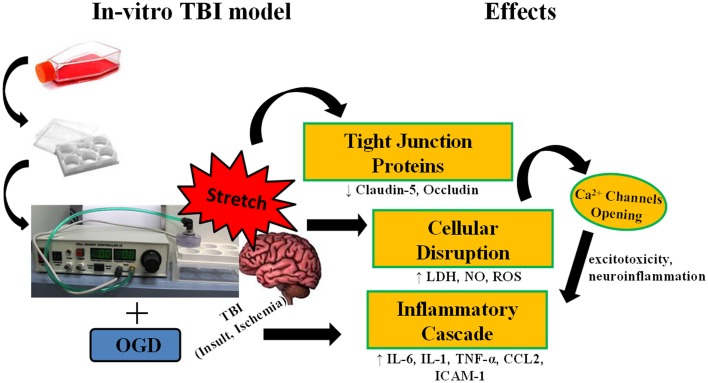
**Stretch induces cellular disruption leading to inflammatory cascade**. Subjecting murine cerebrovascular endothelial cells cEND to stretch, alone, or in combination with oxygen glucose deprivation (OGD) results to compromise of cell membrane integrity. Damage to the cell membrane decreases expression of tight junction proteins. Cellular disruption brings about production of reactive oxygen species (ROS), nitric oxide (NO), and lactate dehydrogenase enzyme (LDH), and could also lead to the opening of calcium ion channels inducing the inflammatory cascade.

### Stretch and OGD alter calcium ion levels in cEND cells and is influenced by glial cells C6

**Figure 10:**
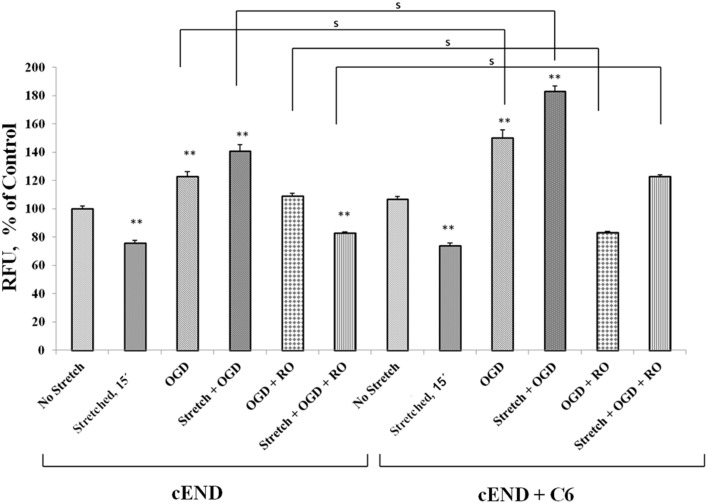
**Stretch and OGD alter calcium ion levels in cEND and cEND + C6 cells**. Calcium concentration relative to that measured in unstretched cEND cells (100%). Values are the percentages of the means (± SEM) of 4 independent experiments. Statistical significance was evaluated using One-Way ANOVA (Holm-Sidak method), ^**^p < 0.001 compared to unstretched cEND cells, s *p* < 0.001. OGD, oxygen glucose deprivation; RO, re-oxygenation.

**Figure 11:**
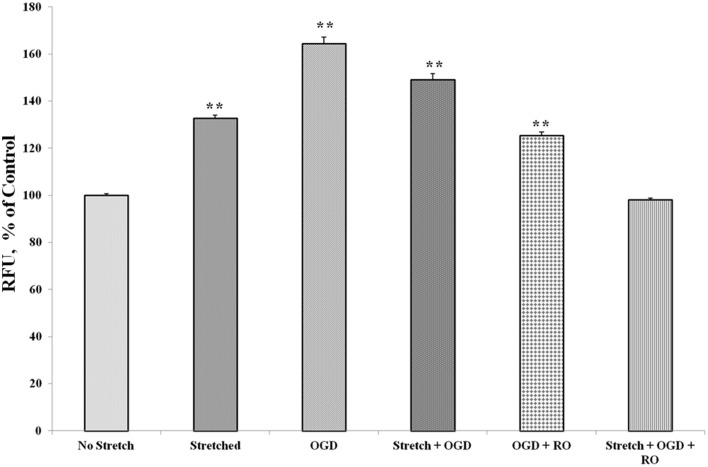
**Stretch and OGD alter calcium ion levels in C6 cells**. Values are the percentages of the means relative to non-stretched C6 cells (± SEM) of 4 independent experiments. Statistical significance was evaluated using One-Way ANOVA (Holm-Sidak method). ^**^p < 0.001 compared to unstretched cEND cells. OGD, oxygen glucose deprivation; RO, re-oxygenation.

Prior to subjecting cEND cells to stretch and/or OGD, glioma cells C6 were subjected to severe stretch and/or 4 h of OGD. Cell culture medium was collected from the C6 cells, added to the cEND cell culture and incubated for 24 h prior to further handling in order to generate interaction between endothelial cells and astrocyte-secreted factors. OGD and a combination of stretch and OGD significantly increased calcium concentration in both cEND cells and cEND cells incubated in medium of C6 cells subjected to stretch or OGD, compared to unstretched cells. On the other hand, stretch significantly decreased calcium concentration in both cEND cells and cEND cells with C6 medium compared to unstretched cEND cells. Incubation of cEND cells with medium from C6 cells subjected to OGD prior to OGD and stretch combined with OGD resulted to significantly higher calcium levels, suggesting influence of astrocytes on cEND cells (Figure 10). Meanwhile, both stretch injury and OGD significantly increased calcium concentration in C6 cells (Figure 11). Addition of the calcium blocker isradipine decreased calcium ion concentration in all samples (Data not shown).

### Stretch and OGD increase permeability of endothelial monolayer

**Figure 12:**
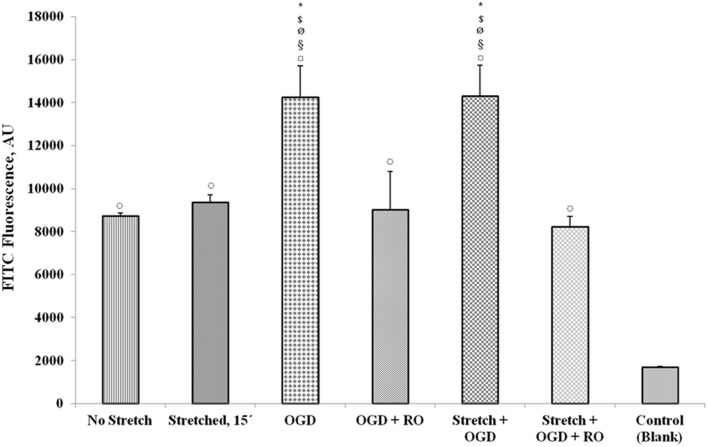
**Measurement of endothelial monolayer permeability under stretch and/or OGD conditions**. The quantification of FITC-labeled avidin bound to the biotinylated gelatin-coated membrane was done to assess permeability of the cell monolayer. FITC fluorescence was measured using a multilabel plate reader. Values are the percentages of the means relative to non-stretched cells (± SEM), *n* = 3. Statistical significance was evaluated using One-Way ANOVA (Holm-Sidak method), ^*^, $, ø, §, ¤ *p* < 0.005 compared to no stretch, stretched 15′, OGD + RO, Stretch + OGD + RO, and control (blank), respectively. OGD, oxygen glucose deprivation; RO, re-oxygenation. Blank control refers to uncoated wells.

Upon subjecting the cells to stretch and/or OGD with or without re-oxygenation, quantification of FITC-labeled avidin bound to the biotinylated gelatin-coated membrane was done to assess permeability. Results show that OGD and a combination of stretch and OGD increased the permeability of the cEND monolayer significantly (Figure 12). Stretch also increased the permeability although not significantly. When the cells were re-oxygenated after OGD and stretch, the permeability reverted to the same level as that of the cells that did not undergo any treatment.

## Discussion

We report the effects of stretch injury as an *in vitro* model for traumatic brain injury (TBI) (Salvador et al., 2013) alone or combined with oxygen glucose deprivation (OGD) in the *in vitro* model of the blood brain barrier (BBB), cerebrovascular brain endothelial cells cEND as well as the expression of glucose transporter GLUT-1 in these cells. OGD has been used to mimic stroke conditions *in vitro* using the brain microvascular endothelial cell cerebEND in combination with astrocytes (Neuhaus et al., 2014). On the other hand, stretch-induced injury has been employed in the murine brain microvascular endothelial cells bEND3 (Berrout et al., 2011). To our knowledge, however, no study that uses a combination of both or comparing the effects of both can be found in the literature and is thus the aim of our study.

TBI is damage to the brain as a result of an external mechanical force i.e., rapid acceleration or deceleration, blast waves, crush, an impact, or penetration by a projectile (Maas et al., 2008). It involves two mechanisms of injury. Primary injury results from the immediate mechanical disruption of brain tissue that occurs at the time of exposure to the external force. It includes contusion, damage to blood vessels and axonal shearing. Meanwhile, secondary injury evolves over minutes to months after the primary injury and is the result of cascades of metabolic, cellular, and molecular events that ultimately lead to brain cell death, tissue damage, and atrophy (Gaetz, 2004; Cernak, 2005). In this paper, stretch was used to simulate the primary mechanical disruption and OGD as a model of ischemia to mimic secondary injury. Inflicting cEND cells with stretch injury was shown to alter their morphology as a result of deformation. As a result of injury, production of cytotoxicity marker lactate dehydrogenase enzyme (LDH), inflammatory markers interleukin (IL)-6, IL-1α, chemokine (C-C motif) ligand 2 (CCL2) and tumor necrosis factor (TNF)-α and total nitric oxide (NO) was induced in the cells. In addition, disruption of cell permeability was shown by microscopy as well as reduction in the amount of tight junction proteins occludin and claudin-5 produced. These parallel some events that occur during primary and secondary injuries in actual TBI (Figure 9).

In our experiments, LDH was significantly increased in the cells subjected to stretch compared to control cells. LDH activity in the culture medium is as an indicator of cell membrane integrity, and thus a measurement of cytotoxicity (Haslam et al., 2000; Wolterbeek and van der Meer, 2005). Therefore, an increase in the amount of LDH released by the cells is tantamount to cell disruption. Initially, our results showed that a greater degree of stretch generated the highest LDH release. For this reason, we used severe stretch in the succeeding experiments. It has already been shown in porcine microvascular endothelial cells that increased stretch injury results to stretch-dependent increase in LDH (Gidday et al., 1999). In the same manner, OGD has been found to generate increased LDH expression in rat endothelial cells (Ceruti et al., 2011). In comparing LDH release among stretch-, OGD- and stretch plus OGD-subjected cells, we observed greater LDH release in all three compared to control. However, stretched as well as stretched and OGD-subjected cells generated more LDH compared to those subjected to OGD. These results indicate injury outcome in both methods used, but in varying degrees.

In as much as cell disruption took effect with the application of stretch, we anticipated an inflammatory cascade. Indeed, our results demonstrated such. For instance, our model exhibited increased IL-6 release in stretched cells after 24 h. Previous studies also proved that to be the case. IL-6, a major inflammatory mediator, is one of the first cytokines produced after TBI. Expression of IL-6 in the brain is low under physiological conditions. However, levels are elevated in a large number of human central nervous system (CNS) disorders (Lucas et al., 2006). After severe TBI, IL-6 is increased in the cerebrospinal fluid (CSF) (Kossmann et al., 1995; Bell et al., 1997). It was reported in a study of canine TBI that control brains showed little expression of inflammatory cytokines in the brain. On the other hand, immunoreactivity for pro-inflammatory cytokines such as IL-1β, IL-6, and TNF-α has been identified in the injured brains. It has also been shown that TBI activates an inflammatory reaction initiated by the release of pro-inflammatory cytokines such as IL-1β, IL-6, and TNF-α (Yu et al., 2011). IL-1 expression is rapidly increased in response to experimental insults such as head injury, cerebral ischemia, seizures, or CNS infections (Mogi et al., 1994; Touzani et al., 1999). In our study, IL-1α was increased in cells subjected to stretch after 24 h post-injury as well as OGD and a combination of both. IL-1 occurs in several forms with IL-1α and IL-1β as the most studied and well-known forms. IL-1α is the predominant form in mice. For this reason, it is the form included in the experiment. IL-1α is a pro-inflammatory cytokine identified to be important in inflammatory response following TBI. It is important in the regulation of the inflammatory response as well as the balance between pro-inflammatory and anti-inflammatory cytokines (Arend, 1991; Dinarello, 1991). IL-1α and IL-1β differ in many ways. IL-1β is secreted and circulates systemically while IL-1α is generally associated with the plasma membrane of the producing cells and acts locally. In addition, IL-1β is mainly produced by monocytes and macrophages while IL-1α is expressed by keratinocytes and endothelial cells (Sims and Smith, 2010). IL-1 triggers inflammatory reactions. This leads to recruitment of leukocytes, disruption of BBB and formation of edema. In addition, it induces the production of other interleukins, prostaglandins, histamine, thromboxane, chemokines and adhesion molecules and exerts multiple effects in neuronal, glial and endothelial cells (Hopkins and Rothwell, 1995; Rothwell and Hopkins, 1995). Similarly, TNF-α was markedly increased in stretched cells 24 h post-injury. Ischemic or traumatic brain injury induces synthesis of TNF-α precursor peptide, pro TNF-α, leading to the release of soluble TNF-α into extracellular space (Shohamia et al., 1999). TNF-α plays a significant role in calcium signaling, which is important for increased excitotoxicity and neuroinflammation. It was found out that TNF-α depresses calcium signal communication in BBB endothelial cells, by reducing gap junctional coupling and by inhibiting triggered ATP release (Vandamme et al., 2004).

NO affects different processes in vascular endothelial cells (Blecharz et al., 2014). Our experiments showed increased NO production after OGD but not stretch. NO accumulates in the brain after injury. It was reported that NO accumulates in the brain after injury in two time periods: immediately after injury and then, several hours-days later. It can be produced by nearly all tissue of the body but physiologically, its expression is found highest in the brain. NO levels are significantly increased but very transiently and then decreased significantly within 30 min of controlled cortical impact (CCI) TBI in rats (Cherian et al., 2004). The production of nitric oxide is catalyzed by nitric oxide synthases (NOS). Three isoforms of NOS exist: the constitutive (neuronal and endothelial) and inducible NOS. Endothelial NOS (eNOS) synthesizes NO only when calcium ion concentration is elevated (Förstermann et al., 1998). Brain ischemia triggers a cascade of events leading to the activation of Ca^2+^-dependent NOS isoforms. Nitric oxide biosynthesis is a key factor in the pathophysiological response of the brain to hypoxia-ischemia (Bolaños and Almeida, 1999). Stretch injury in mouse brain microvessel endothelial cells (bEnd3) has also been shown to increase calcium influx and NO production (Berrout et al., 2011). TBI alters NO synthesis rates and brain tissue NO levels (Ahn et al., 2004). It is an endothelium-relaxing factor and a cerebral and systemic vasodilator (Faraci et al., 1994). It contributes to a constant state of partial dilation. Therefore, an increase in NO production would increase cerebral blood flow after TBI. In the ischemic cerebral cortex, NO production was observed to have rapidly and transiently increased after focal cerebral ischemia (Kader et al., 1993).

Our *in vitro* model of the blood brain barrier, cEND, demonstrates characteristics of a suitable model of the BBB (Förster et al., 2005). The BBB is formed by brain capillary endothelial cells with tight junctions (TJs) as its most important structural components (Rubin and Staddon, 1999). Claudin-5 is one of the TJ proteins critical in sealing the paracellular space of the BBB (D'Atri and Citi, 2002). It plays an important role in the permeability of the blood brain barrier. Hence, its degradation could result in BBB breakdown (Förster, 2008). OGD reduced the expression of TJ proteins and transendothelial resistance in cEND cells (Kleinschnitz et al., 2011). This could also be depicted in our use of stretch and OGD with decreased protein expressions of claudin-5 and occludin.

Permeability is an important measure of BBB integrity (Audus et al., 1990). During injury when the BBB is disrupted, the permeability of the endothelium is increased. It is thus important to investigate the functionality of the endothelial monolayer in an *in vitro* model system that targets to reproduce the events that occur *in vivo* during TBI and ischemia. The increased permeability of the endothelial monolayer during stretch and OGD in our *in vitro* model demonstrates BBB disruption. The tight junctions present in intact endothelium limit the diffusion of molecules between the cells and the low levels of pinocytic activity restrict the intracellular transport of molecules (Audus and Borchardt, 1991). Under certain conditions such as injury, the low permeability state of the BBB is altered, allowing the influx of macromolecules which could lead to neuronal damage.

During TBI, there is massive influx of calcium ions into the cells of the CNS. It has been shown that total cell calcium increases in astrocytes after injury (Hovda et al., 1992; Fineman et al., 1993; Rzigalinski et al., 1997). In our experiments, stretch and OGD as well as a combination of both, increased calcium ion levels in C6 cells. A reversion to levels similar to that of control was observed after re-oxygenation and re-supplementation with glucose. This is in accordance to published results that the levels of calcium concentration in astrocytes increase after stretch injury and return to normal levels by 15 min post-injury (Rzigalinski et al., 1998). The same has been observed for astrocytes that were subjected to ischemic injury (Cheung et al., 1986; Choi, 1988). Meanwhile, it has been reported that mechanical injury of brain endothelial cells induces a rapid influx of calcium immediately after injury. However, the increase was only small and transient in the absence of extracellular calcium as opposed to when cells were supplied with extracellular calcium (Berrout et al., 2011). This suggests that the increase in calcium levels is due to calcium influx and not calcium release. In our experiments, the levels of calcium ions decreased in cEND cells after stretch, followed by 15 min recovery. The amount of time wherein the cells where made to recover possibly contributed to the decreased calcium level. Activation of tyrosine kinase receptors results to calcium influx in bovine aortic endothelial cells (Munaron and Fiorio Pla, 2000). Meanwhile, reactive astrocytes were shown to express functional tyrosine kinase receptors following insult to the cerebral cortex (McKeon et al., 1997) which could be the reason for increased calcium concentration in the cEND cells incubated in medium of injured C6 cells compared to cEND cells alone.

Traumatic brain injury (TBI) involves a cascade of morphological and physiological events. Most *in vivo* models of TBI meet the criteria of reproducing such occurrences (Morales et al., 2005; Albert-Weissenberger and Sirén, 2010). In order for an *in vitro* system to be suitable as a model, it should also be able to mimic these incidents (Kumaria and Tolias, 2008; Morrison et al., 2011). Here, we report the effects of stretch alone or in combination with OGD to an existing *in vitro* blood-brain barrier model (BBB), cEND. The use of both stretch and OGD leads to cellular disruption, decrease in tight junction proteins and increase inflammatory markers expression. Overall, the effects of OGD appear to be more prominent than that of stretch. OGD decreased expression of tight junction proteins claudin-5 and occludin, and increased expression of NO and glucose transporter GLUT-1 more significantly than stretch. Nonetheless, there are specific effects brought about by stretch such as a marked increase in TNF-α expression which is not the case for OGD. Therefore, a combination of both enhances the replication of events in TBI, an important feature sought for in an *in vitro* TBI model.

### Conflict of interest statement

The authors declare that the research was conducted in the absence of any commercial or financial relationships that could be construed as a potential conflict of interest.
